# Vitamin D deficiency and its characteristics among patients with acute stroke at a national referral hospital in Kampala Uganda

**DOI:** 10.1186/s12902-015-0053-y

**Published:** 2015-10-05

**Authors:** Daniel S. Kiggundu, Edrisa Mutebi, Davis Kibirige, Rebecca Boxer, Barbara Kakande, Brian K. Kigozi, Elly Katabira

**Affiliations:** Department of Medicine, College of Health Sciences, Makerere University, Kampala, Uganda; Department of Medicine, Uganda Martyrs Hospital, Lubaga, Kampala, Uganda; Department of Medicine, Case Western Reserve University, Cleveland, OH USA; Uganda Heart Institute, Mulago Hospital, Kampala, Uganda; Uganda Virus Research Institute, Entebbe, Uganda

**Keywords:** Vitamin D deficiency, Stroke, Uganda

## Abstract

**Background:**

Vitamin D deficiency has been associated with acute stroke and other cardiovascular diseases in the developed world. Low 25-hydroxyvitamin D (25OHD) has been described in some populations in Sub-Saharan Africa (SSA) in spite of adequate sunshine all year round. There is no information on the magnitude of vitamin D deficiency among patients with stroke and other cardiovascular diseases in Uganda or SSA. The aim of this study was to determine the burden and characteristics of vitamin D deficiency among patients with acute stroke, the most common form of cardiovascular events in SSA.

**Methods:**

We conducted a cross-sectional study between October 2012 and March 2013. We consecutively recruited 142 subjects with acute stroke admitted to the medical wards of Mulago hospital. We administered a pre-tested questionnaire to the study participants, and did a detailed physical examination and laboratory evaluation. Serum levels of 25OHD were determined using an electrochemiluminescence assay. Data were analyzed using STATA version 12 software.

**Results:**

The prevalence of vitamin D deficiency (25OHD < 20 ng/ml) was 15 %. Longer hours of sunshine exposure decreased the likelihood vitamin D deficiency significantly (adjusted OR 0.85, *p* = 0.03). Higher HDL cholesterol had a significant inverse association with vitamin D deficiency (adjusted OR 0.15, *p* = 0.02). In addition, the likelihood of vitamin D deficiency increased with rising age (adjusted OR 1.03, *p* = 0.05).

**Conclusions:**

There was a relatively low burden of vitamin D deficiency among patients with acute stroke in Uganda. With increasing longevity and indoor lifestyles vitamin D deficiency may assume a greater role in stroke and other cardiovascular diseases in tropical sub Saharan Africa. Future studies on the mechanisms of vitamin D deficiency and its relationship to outcomes among patients with stroke may be necessary.

**Electronic supplementary material:**

The online version of this article (doi:10.1186/s12902-015-0053-y) contains supplementary material, which is available to authorized users.

## Background

Vitamin D deficiency is a widespread health problem, affecting up to 1 billion people worldwide [1]. It has gained increasing recognition in the developing world, including Sub- Saharan Africa (SSA) where sunshine prevails all year round [2–4]. Previous studies in SSA have found widely varying prevalence of vitamin D deficiency in a tropical region with good quality UVB radiation [5–8]. For example, studies in Uganda which straddles the equator have reported a prevalence of vitamin D deficiency ranging between 9–20 % among healthy individuals [5, 6]. In central Ethiopia (8^0^ 33 N to 8^0^ 36 N) a prevalence of up to 61 % has been reported among otherwise healthy urban schoolchildren [8]. Latitude, season, cultural norms, religious practices, low awareness, low knowledge/health literacy, indoor lifestyles, urban living, skin pigmentation, malnutrition, diet, co-morbidities like tuberculosis, and drugs may contribute to vitamin D deficiency in the SSA region [2, 3, 9, 10].

Classically, vitamin D deficiency is known for its effects on bone, causing reduced mineralization, rickets and osteomalacia [1, 3]. However, many new roles of vitamin D have recently come to light, with more consequences attributed to its deficiency [1]. Vitamin D deficiency is increasingly associated with infectious diseases like tuberculosis and severe malaria [6, 11, 12], and non communicable diseases such as cancer, depression, dementia, multiple sclerosis, cardiovascular diseases, and stroke [1, 6, 13–16].

Cardiovascular diseases are on the rise in SSA, where they are projected to become the leading cause of morbidity by 2030 [17]. Stroke contributes the biggest proportion of cardiovascular disease in SSA and it is responsible for up to 3 % of all adult deaths [17, 18].

Studies in non-tropical settings have described higher burden of vitamin D deficiency among acute stroke patients than non- stroke individuals in the same environment [16, 19]. Suboptimal vitamin D has been associated with many modifiable cardiovascular risk factors, including physical inactivity, hypertension, dyslipidemia, obesity and diabetes mellitus, including among migrant populations from Sub Saharan Africa [13, 15, 20–23]. Furthermore, longitudinal studies have demonstrated that vitamin D is an independent predictor of cardiovascular disease and stroke [13, 19]. The relationship of vitamin D deficiency to large vessel stroke and other atherosclerotic cardiovascular diseases may be mediated by atherogenic proinflammatory cytokines that foster atherosclerotic vascular changes and plaque instability [24, 25].

Among stroke patients, vitamin D deficiency has been shown to predict severity and adverse outcomes including death [19, 26]. Stroke survivors with vitamin D deficiency may be prone to bone loss and pathological fractures [16, 26]. Supplementation with oral 25OHD has been suggested as a potential strategy to reduce cardiovascular diseases and other non-communicable diseases [27]. Ongoing large scale clinical studies in North America are evaluating the role of 25OHD replacement in the prevention of stroke among other non communicable diseases [28].

Currently, there are few data on the magnitude of vitamin D deficiency in patients with cardiovascular diseases in tropical SSA with its unique latitude, indigenous peoples and culture. We therefore sought to determine the prevalence and characteristics of vitamin D deficiency in hospitalized patients with acute stroke at Mulago national referral and teaching hospital (Mulago hospital) in Kampala, Uganda.

## Methods

### Study design and site

This was a cross- sectional study conducted on the medical wards of Mulago hospital from October 2012 to March 2013. The medical wards receive 20–30 stroke patients on the neurology service monthly [29]. Mulago hospital is a 1600 bed- capacity hospital and it is the largest referral hospital in Uganda. Uganda is located on the East Africa plateau and spans the equator from approximately 1°S to 4°N. Kampala city is located near the equator at latitude of 00°20′North, with constant sunshine all year round and two rainy seasons; one from March to May and the other between September and December.

### Study definitions and participants

For the purposes of this study, acute stroke was defined as a stroke that had lasted not more than 2 weeks from the onset of symptoms. Stroke diagnosis was made as per the classical World Health Organisation definition that identifies stroke as ‘a clinical syndrome typified by rapidly developing signs of focal or global disturbance of cerebral functions, lasting more than 24 h or leading to death, with no apparent causes other than of vascular origin’. Patients were included with or without radiological confirmation using brain computerised tomography (CT) scan.

A period within 2 weeks of symptoms was used to designate acute stroke, in order to reflect pre-stroke concentrations of 25OHD, with minimal post-stroke influence. This was chosen because 25OHD has a half life of 2–3 weeks,depending on 25OHD type and concentration, vitamin D binding protein genotype and concentration, and race [30].

The participants’ 25OHD status was defined basing on the Endocrine Society Clinical Practice Guidelines on evaluation, treatment and prevention of vitamin D deficiency [31].

Normal 25OHD level was defined as serum 25OHD ≥ 30 ng/ml. Vitamin D insufficiency was defined as serum 25OHD 20- < 30 ng/ml. Vitamin D deficiency was used to describe 25OHD <20 ng/ml. That included severe vitamin D deficiency with 25OHD <10 ng/ml.

During the study period, consecutive sampling was used to approach all adults (aged ≥18 years) admitted to the medical wards with a diagnosis of acute stroke made by the ward team. Consent was obtained from patients or their relatives, and patients who fulfilled the study definition of acute stroke were consecutively enrolled into the study. Patients for whom no history could be obtained were excluded from the study.

### Data collection

After obtaining informed consent, pre-tested questionnaires were administered by the study team to obtain the socio-demographic characteristics, medical history (history of hypertension, diabetes mellitus, HIV), drug history (anti-convulsants, anti-retroviral therapy, multi-vitamin supplements, and anti tuberculous drugs), exercise and estimated duration spent outdoors on a typical day. HIV status was determined using a routine testing and counselling algorithm of Mulago Hospital.

Diabetes mellitus (DM) status was decided by obtaining history of a previous diagnosis of diabetes mellitus, or a random glucose measurement ≥11.1 mmol/L. History of a previous diagnosis of hypertension was obtained. Blood pressure was measured using the Omron M2 Eco digital blood pressure machine. Where the attending clinical team reported a diagnosis of hypertension based on additional clinical findings, it was accepted as part of the history. However, due to the possibility of reactive changes in blood pressure during acute stroke, blood pressure measurements were not used to define hypertension. Lipid profiles and blood glucose were measured without strict observance of fasting state due to practical challenges in enforcing fasting among often severely ill patients.

Waist circumference was measured to assess abdominal obesity using the International Diabetes Federation (IDF) criteria [32]. The participants’ Glasgow Coma Scale (GCS) score and stroke type as identified on brain CT scan were also documented (Additional file 1).

### Laboratory assessment

#### Sample collection

From each participant, a blood sample (5 ml) was drawn aseptically into a plain blood sample tube (BD Vacutainer) and allowed to coagulate at room temperature.

The blood samples were transported to the clinical chemistry laboratory of Mulago hospital within 1 h of collection, centrifuged and the extracted sera stored at −20 °C in a refrigerator until testing.

#### Diagnostic testing

Chemistry analysis was done on thawed sera, using the fully automated COBAS® 6000 (Roche Diagnostics GmbH) machine. Serum 25OHD concentration was determined using an electrochemilumniscence immunoassay: the Elecsys Vitamin D Total assay (Roche diagnostics, Mannheim-Germany; Reference number 05894913190 V4). It measures total serum 25OHD concentrations in the range of 3–70 ng/ml. The COBAS 6000 analyzer was also used to measure the serum lipids.

Measurement of albumin, calcium concentration, creatinine and lipid profile was also done. (Lipid profile included low density lipoprotein cholesterol [LDLC] and high density lipoprotein cholesterol [HDLC].

### Statistical analysis

Based on the prevalence of vitamin D deficiency (defined as vitamin D levels < 20 ng/ml) of 9 % among healthy individuals in a study in Mulago Hospital, Kampala [5] with a 95 % CI and desired precision of estimation of 0.05, a sample size of 126 patients was obtained. We enrolled 142 patients consecutively over a 6 months study period; however, due to logistical constraints we did not adjust sample size for associated factors.

Data were entered using Epi data entry version 3.1 and exported to Stata version 12.1 software, for statistical analysis. Patient characteristics were reported in tables as proportions. Continuous variables were reported as median and inter-quartile range (IQR). Relationship of 25OHD and continuous variables was explored with Pearsons correlation coefficient.

With the study goal to identify characteristics associated with vitamin D deficiency, factors were entered into a logistic regression model. All statistically significant factors from the univariate analysis with *p* < 0.1 were entered into the multivariable logistic regression model. In addition, categorical outcomes that were not binary were considered for inclusion if they had a significant overall main effect. Important confounder variables including gender, diabetes mellitus, central obesity and a history of hypertension were also included in the model by a priori decision regardless of statistical significance. A *p*-value of <0.05 and confidence intervals not including 1 were considered to be statistically significant at multivariable analysis.

### Study approval

The study was approved by the Makerere University College of Health Sciences, School of Medicine Research and Ethics Committee. For all patients, written informed consent was obtained prior to enrolment in the study from their relatives or care takers. All participants were given information on the positive effect of exposure to sunshine on 25OHD levels. Where vitamin D deficiency was found, the result was explained to the patient/caretaker by telephone, with a recommendation to increase sunshine exposure and/or oral 25OHD supplementation. The result was made available to the medical clinic at routine follow up visits.

## Results

### Socio-demographic characteristics

Of the 168 patients screened, 142 patients were enrolled consecutively. Among the 26 ineligible patients, an alternative diagnosis was found in 12 patients and no reliable source of history was available for 12 others. For 2 patients, informed consent was not given. The response rate was 91 % (142 of 156 patients with stroke). The overall median age (interquartile range (IQR) of patients was 69 (50–70) years; predominantly females, 54 %; residing in urban and peri-urban areas, 62 %; and had attended primary education, 61 % (Table 1). The median duration of symptoms at enrolment was 5 days (IQR 3–7), and most patients (90 % of 142) were enrolled within 10 days of onset of the symptoms.

**Table 1:** Clinical characteristics associated with vitamin D deficiency among in-patients with acute stroke in Kampala Uganda- bivariate analysis

Variable	Total N (% of total) or median (IQR)	25OHD <20 ng/ml n (% of row) or median (IQR)	Crude odds ratio (OR)	95 % confidence interval	*P* value
Gender					
Male	69 (49)	12 (17)	1.00		
Female	73 (51)	9 (12)	0.67	0.27–1.70	0.40
Hypertension history					
No	67 (47)	7 (10)	1.00		
Yes	75 (53)	14 (19)	1.97	0.74–5.21	0.17
Diabetes mellitus					
No	117 (82)	18 (15)	1.00		
Yes	25 (18)	3 (12)	0.73	0.20–2.72	0.64
HIV status					
Negative	130 (92)	19 (15)	1.00		
Positive	12 (8)	2 (17)	1.16	0.24–5.75	0.85
Previous Stroke					
No	127 (89)	15 (12)	1.00		
Yes	15 (11)	6 (40)	4.98	1.55–15.96	0.01
Typical dress					
Gomesi^a^	48 (34)	5 (10)	1.00		
Short sleeve + trouser	33 (23)	7 (21)	2.32	0.67–8.05	0.19
Long sleeve + trouser	28 (20)	4 (14)	1.43	0.35–5.84	0.62
Other	33 (23)	5 (15)	1.54	0.41–5.79	0.53
Obesity					
No	81 (57)	13 (16)	1.00		
Yes	61 (43)	8 (14)	0.79	0.30–2.04	0.63
Stroke type					
Ischemic	93 (76)	17 (18)	1.00		
Hemorrhagic	30 (24)	2 (7)	0.32	0.27–1.70	0.14
^b^Age (years)	69 (50–70)	70 (62–79)	1.03	1.00–1.06	0.08
Daily time outdoors (h)	6.75 (3–10)	3 (1.5–7.5)	0.85	0.75–0.96	0.02
Exercise (days per week)	1 (0–7)	1 (0–7)	0.95	0.82–1.10	0.51
Systolic BP (mmHg)	155 (139–171.5)	150 (141–170)	1.00	0.98–1.01	0.58
Diastolic BP (mmHg)	90 (77–101.5)	90 (80–103)	1.00	0.98–1.01	0.71
Corrected Ca^++^ (mmol/l)	2.32 (2.22–2.39)	2.31 (2.23–2.36)	0.21	0.01–5.38	0.34
Random glucose (mmol/l)	6.7 (5.7–8.6)	6.7 (5.9–7.1)	1.09	0.91–1.30	0.33
Albumin (g/L)	37.4 (33.5–41.0)	36.6 (30.5–40.6)	0.93	0.86–1.01	0.07
Total cholesterol (mmol/L)	4.53 (3.59–5.45)	4.58 (3.18–5.4)	0.89	0.63–1.24	0.49
HDL cholesterol (mmol/L)	1.18 (0.94–1.43)	0.58 (1.31–1.15)	0.25	0.07–0.92	0.04

^a^Uganda national women’s dress covering lower limbs and torso leaving arms bare

^b^Data from this row onward are continuous; association with the categorical outcome of vitamin D deficiency is displayed as odds ratios

### Clinical and laboratory findings

A previous history of hypertension was obtained in 75 patients (53 % of 142). Diabetes mellitus was found in 25 patients (19 % of 142); 19 patients by history of DM, and an additional 6 patients based on a high random blood glucose measurement. Other co-existing co-morbidities were HIV infection, non-atrial fibrillation cardiac disease, atrial fibrillation and chronic kidney disease in 9, 6, 4 and 3 % of the patients respectively. One patient had sickle cell disease and one patient had tuberculosis.

Antiretroviral therapy was used by 5 of the 12 HIV positive subjects, with majority (60 %) using an efavirenz based regimen. The use of multivitamin supplements was not found in any patient.

Prior history of stroke was reported in 15 (11 %) patients. A brain CT scan was done in 123 patients (87 % of 142). However, 19 patients (13 % of 142) did not get brain CT scans due to financial constraints. Ischemic stroke was the most prevalent type of stroke, noted in 93 (76 %) patients.

The median HDL Cholesterol (HDLC) and total cholesterol (TC) in mmol/l was 1.18 (0.94–1.43), and 4.35 (3.59–5.45) respectively. The median calcium concentration, corrected for albumin levels in was 2.32 mmol/l (1.99–2.59)

### 25OHD status and related factors

The prevalence of vitamin D deficiency was 15 % (21 out of 142 patients). This included 5 patients (4 % of 142) who were categorized as having severe vitamin D deficiency. Vitamin D insufficiency (25OHD 20- < 30 ng/ml) was found in 29 patients (20 % of 142). Overall two thirds (65 % of 142) of the study patients had adequate vitamin D levels. The median (IQR) concentration of 25OHD was 36.4 ng/ml (22.0–45.7).

The burden of vitamin D deficiency was greater among patients with ischemic stroke than in patients with hemorrhagic strokes, although not statistically significant (18 % vs. 7 %, *p* = 0.33). An age adjusted prevalence of vitamin D deficiency by stroke type (computed using the age distribution of the entire sample) was similar to the crude prevalence, at 19 % versus 9 % in patients with ischemic and hemorrhagic strokes respectively.

Among subjects with vitamin D deficiency, there were more patients with a history of hypertension (*n* = 14, 67 % of 21) and more male patients (*n* = 12, 57 % of 21). Vitamin D deficiency was found in 6 (29 % of 15) patients with a previous history of stroke, and 2 (17 % of 12) patients with HIV. None of the patients on antiretroviral therapy had vitamin D deficiency.

No significant linear relationship was observed between 25OHD and systolic blood pressure (*r* = −0.04, *p* = 0.62), 25OHD and diastolic blood pressure (*r* = −0.05, *p* = 0.58), or 25OHD and random blood glucose (*r* = −0.03, *p* = 0.69).

At bivariate analysis, increasing age,, prior history of a stroke, hours spent outdoors, increasing albumin and HDLC concentrations had *p* values ≤ 0.1 and were entered into the multivariate analysis model, Gender, diabetes mellitus, obesity, and a history of hypertension were also included as important confounders (Table 2). Lower HDLC concentrations (OR 0.09, *p* = 0.02) and shorter duration outdoors (OR 0.83, *p* = 0.02) were independently associated with vitamin D deficiency among stroke patients in the multivariate analysis model. A previous history of stroke (OR 3.8, *p* = 0.06) and increasing age (OR 1.04, *p* = 0.05) were not significantly associated with vitamin D deficiency.

**Table 2:** Clinical characteristics associated with vitamin D deficiency among in-patients with acute stroke in Kampala Uganda-multivariate analysis

Variable	Crude odds ratio	Adjusted odds ratio	95 % CI	*P* value
Gender				
Male	1.00			
Female	0.67	1.18	0.38–3.67	0.77
Diabetes				
No	1.00			
Yes	0.73	0.29	0.41–2.10	0.22
Obesity				
No	1.00			
Yes	0.79	0.71	0.21–2.37	0.57
Hypertension history				
No	1.00			
Yes	1.97	2.00	0.62–6.43	0.25
Previous stroke				
No	1.00			
Yes	4.98	3.80	0.94–15.32	0.06
^a^Age(years)	1.03	1.04	1.00–1.08	0.05
Daily time outdoors (h)	0.85	0.83	0.71–0.97	0.02
Albumin (g/L)	0.93	1.01	0.90–1.13	0.92
HDL cholesterol	0.25	0.09	0.01–0.68	0.02

^a^From this row onwards, continuous predictor variables are displayed, with odds ratio of vitamin D deficiency per unit rise in the predictor variable

## Discussion

In this study, we found a relatively low prevalence of vitamin D deficiency among adult in-patients with acute stroke at a referral hospital in East Africa. The significant factors associated with vitamin D deficiency in this group were a lower reported duration of sunshine exposure and lower HDL cholesterol. To our knowledge, this study is the first in SSA to describe the burden of vitamin D deficiency among patients with acute stroke or other cardiovascular diseases.

Comparing studies that report vitamin D deficiency requires attention to the 25OHD cut-off values used to define it. Different cut-off values have been used among studies in East African and elsewhere, ranging from <10 ng/ml to <30 ng/ml [6, 31, 33, 34]. The various cut-off values reflect the changing understanding of the roles of 25OHD beyond only bone health, to include roles in immunology and infections, mental illness, cardiovascular prevention, cancer, and other health outcomes [1, 12, 24, 25, 33, 35]. A cut-off value of <20 ng/ml is widely accepted through consensus among experts [1, 31, 33]. It is also frequently reported even by studies that define vitamin D deficiency otherwise. For purposes of this study, we took care to compare 25OHD <20 ng in reference to vitamin D deficiency.

Our findings on the prevalence of vitamin D deficiency are similar to those in other studies in the region of SSA: in Uganda this prevalence has been 9–20 % among normal study subjects [5, 6]. Among in-patients on a medical ward in Malawi a prevalence of 14 % was found [36]. However, an examination of data from other studies reveals wide contrasts in the SSA region: among traditionally living populations in East Africa have reported a high mean 25OHD value with virtually no vitamin D deficiency [37]. In contrast, studies in central Ethiopia and Northern Nigeria have reported high prevalence of suboptimal 25OHD levels [7, 8].

We found that lower sunshine exposure was associated with vitamin D deficiency in this study. This is expected since ultraviolet B (UVB) radiation in sunshine drives cutaneous vitamin D synthesis [1]. Estimating exposure to sunshine using a questionnaire to establish typical intervals spent outdoors is a method that has been used with some success, but it is limited by recall bias and variation in behaviour during time spent outdoors [38].

The association between older age and vitamin D deficiency was not statistically significant, probably due to the limited sample size and study power. An association between rising age and vitamin D deficiency was the expected; it is biologically plausible due to a decline in the efficiency of cutaneous vitamin D synthesis with increasing age [1, 39]. Paradoxically, another recent study in an urban population in East Africa reported lower levels of 25OHD among younger adults compared to older adults [40]. This may reflect changing lifestyles and decreased sunshine exposure among younger urban populations.

Other factors including, religion, cultural practices, diet, co-morbidities and low awareness may contribute to disparities in the prevalence of vitamin D deficiency seen in the tropics [2, 3]. Genotype of vitamin D binding protein and the type of 25OHD (25OHD2 or 25OHD3) are linked to changes in the serum half life of vitamin D deficiency and the likelihood of vitamin D deficiency [30].

Outside the tropics, studies among patients with acute stroke/cardiovascular events generally report greater prevalence of vitamin D deficiency than we found. In Europe and South East Asia, studies also report a greater burden of vitamin D deficiency among stroke patients compared to control subjects in the same environment [16, 41]. Our study design did not include similar control subjects, limiting our ability to make meaningful comparisons.

Among patients with stroke vitamin D deficiency is reported to predict greater severity and adverse outcomes including recurrent strokes and death [26, 42]. Vitamin D deficiency is also linked with a greater likelihood of falls, poor muscle and bone health, and possibly increased fracture risk among stroke survivors [16, 43]. On the other hand stroke predisposes to vitamin D deficiency, possibly through reduced dietary intake, inability to feed independently, and decreased mobility and sunshine exposure [16, 43]. As expected, we found a higher burden of vitamin D deficiency among the subjects who had suffered at least one previous stroke. Nonetheless, no significant association was found after adjusting for other factors perhaps due to low study power. However, we did not examine the relation of 25OHD status with stroke severity or mortality in our setting. Routine strategies to prevent vitamin D deficiency among stroke survivors in our setting are required.

While vitamin D deficiency is an independent predictor for stroke and other cardiovascular events, it is also associated with traditional risk factors like hypertension, insulin resistance/diabetes, obesity, abnormal lipid profile [24, 44]. Our study indeed showed that the likelihood of vitamin D deficiency increased with a declining HDL level. Studies among immigrants from SSA in Australia have documented vitamin D deficiency clustered with obesity, insulin resistance and dyslipidemia [23], and go ahead to propose a role for vitamin D replacement in cardiovascular prevention [27]. In addition, vitamin D deficiency is related to novel cardiovascular risk factors such as C-Reactive Protein and pro-inflammatory cytokines [24, 44]. We were not able to demonstrate an association between vitamin D deficiency and hypertension-the single most powerful cardiovascular risk factor in SSA today [18].

Importantly the diagnosis of hypertension and diabetes in our study were influenced by low awareness of either condition, physiological responses of blood pressure and blood sugar to acute stroke [45], and absence of supporting tests like the HBA1c assay.

In this region with rising incidence of cardiovascular risk factors and diseases, vitamin D deficiency appears to contribute a minor fraction to the burden at present. The burden of vitamin D deficiency in relation to stroke/cardiovascular diseases may increase with greater longevity, and behavioural change including lower exposure to sunshine.

### Limitations

Due to logistical constraints, low numbers were recruited in our study, thus limiting the power of the study. The diagnosis of acute stroke was made based on clinical history and physical exam findings within 2 weeks of onset, with or without corroborating imaging studies. While this could limit the accuracy of stroke diagnosis, similar approaches have been used in other studies on stroke and vitamin D deficiency. We included all stroke subtypes in this study. Previous studies have not always made a distinction between hemorrhagic and ischemic strokes. Mechanisms by which vitamin D deficiency is related to stroke relate more to atherosclerosis and ischemic stroke.

Different 25OHD assays were used in the studies we have compared, but the new Roche vitamin D total assay that we used has demonstrated strong correlation with high performance liquid chromatography/mass spectrometry and established radioimmunoassay techniques [46]. Due to our study design, we are unable to show a temporal relationship between vitamin D deficiency and stroke events. However, studies elsewhere have frequently found that vitamin D deficiency is associated with stroke, and may indeed predict stroke [19]. Ongoing studies elsewhere are investigating the role of 25OHD replacement on non communicable diseases including cardiovascular disease, cerebrovascular disease and cancer [28].

## Conclusions

There was a relatively low burden of vitamin D deficiency among patients with acute stroke in Uganda. Vitamin D deficiency plays a minor role in stroke risk in Uganda at present. With increasing longevity and indoor lifestyles vitamin D deficiency may assume a greater role in stroke and other cardiovascular diseases in tropical sub Saharan Africa. Future studies may be necessary to evaluate the relationship of vitamin D deficiency to stroke outcomes and the mechanisms thereof.

## Additional file

Additional file 1:
**Study data Collection form.** (PDF 83 kb)
