# Supplementary material for: Vitamin D deficiency and its characteristics among patients with acute stroke at a national referral hospital in Kampala Uganda
Source: BMC Endocr Disord. 2015 Oct 5;15:53. doi: 10.1186/s12902-015-0053-y (PMC4594645; doi:10.1186/s12902-015-0053-y)
Supplement: Additional file 1: — Study data Collection form. (PDF 83 kb) [file 12902_2015_53_MOESM1_ESM.pdf]

**Vitamin D deficiency and its characteristics among patients with acute stroke at a national referral hospital in Kampala Uganda** MS: 8181168691274465

Appendix1: Data Collection form

**Study number** [ ]

## 1 Sociodemographics

| | | | | |
|---|---|---|---|---|
| **1. Age** | | **DOB** | | |
| **2. Sex** | | | 1 female | 2 male |
| **3. Residence** | 1Urban | 2Periurban | 3Rural | |
| **4. Religion** | 1Catholic | 2Protestant | 3.Moslem | 4.Other |
| **5. Tribe** | | | | |
| **6. Occupation** | | | | |
| **7. Education** | 1.None | 2. Primary | 3. Secondary | 4.Tertiary |
| **8. Onset Date** | | | | |

## 2. Stroke Diagnosis

### 2.1 Stroke symptoms (Tick all that apply)

| | | |
|---|---|---|
| 1Limb weakness | 4.Reduced consciousness | 7. Other(specify) |
| 2.Facial weakness | 5.Dizziness | |
| 3.Altered speech | 6.Abnormal movements | |

### 2.2 Stroke Signs (Tick all that apply

| | | | | |
|---|---|---|---|---|
| 1. Limb weakness | Right upper | Right lower | Left upper | Left lower |
| 2. Facial weakness | | | | |
| 3. Other Cranial nerve palsy | | | | |
| 4. Abnormal | | | | |
| 5. Abnormal tone | | | | |
| 6. GCS score | E | V | M | Total |
| Other (specify) | | | | |

### Imaging

| | | |
|---|---|---|
| Brain CT done | 1.Yes | 2.No |
| If yes, subtype | 1.Ischemic | 2.Hemorrhagic |

## 3. Previous Medical history conditions

| | | | |
|---|---|---|---|
| Hypertension | | 1 yes | 2. no |
| Diabetes | | 1 yes | 2.no |
| HIV | | 1 Positive | 2 negative |
| Other (specify) | | | |

## 4. Drug history

| | | |
|---|---|---|
| Antiretroviral therapy | 1. Yes (specify) | 2. No |
| Azole Antifungal | 1. Yes (specify) | 2. No |
| Anticonvulsants | 1. Yes (specify) | 2. No |
| Corticosteroids | 1. Yes (specify) | 2. No |
| Other (specify) | | |

## 5. Lifestyle measures

How many days a week do you walk for ≥30 minutes?

| | | |
|---|---|---|
| | | |

## 6. On a typical day, how much time (minutes) do you spend outdoors

| | |
|---|---|
| 6am-10am | |
| 10am-4pm | |
| 4pm-7pm | |

## 7. Mode of dress on a typical day.

| |
|---|
| |

## 8. Physical examination

| | |
|---|---|
| Weight (kg) | |
| Height/length (m) | |
| Waist circumference (cm) | |
| Hip circumference (cm) | |
| Pulse rhythm | |
| Blood pressure mmHg | |

## 9. Lab measurements

| | | | |
|---|---|---|---|
| 25hydroxy-vitamin | | Total cholesterol | |
| RBG | | HDL | |
| Serum albumin | | LDL | |
| Serum calcium | | Non HDL | |
| Serum creatinine | | HIV screen | |

**Interviewed By** **Date of interview**
